# Calls to the anti-violence number in Italy during COVID-19 pandemic: correlation and trend analyses of violence reports during 2020

**DOI:** 10.1007/s00127-022-02330-x

**Published:** 2022-07-08

**Authors:** Antonio Del Casale, Martina Nicole Modesti, Carlo Lai, Chiara Ciacchella, Giorgio Veneziani, Benedetta Barchielli, Stefano Ferracuti, Christian Napoli, Maurizio Pompili

**Affiliations:** 1 Department of Dynamic and Clinical Psychology, and Health Studies, Faculty of Medicine and Psychology, Sapienza University, Rome, Italy; 2 Department of Neuroscience, Mental Health and Sensory Organs (NESMOS), Faculty of Medicine and Psychology, Sapienza University, Rome, Italy; 3 Department of Human Neuroscience, Faculty of Medicine and Dentistry, Sapienza University, Rome, Italy; 4 Department of Medical-Surgical Sciences and Translational Medicine, Faculty of Medicine and Psychology, Sapienza University, Rome, Italy; 5 Unit of Psychiatry, ‘Sant’Andrea’ University Hospital, Via di Grottarossa 1035-1039, 00189 Rome, Italy

**Keywords:** COVID-19 pandemic, Gender violence, Quarantine, Family violence, Mental health

## Abstract

**Purpose:**

We hypothesized that during the 2020 pandemic there has been a significant change along the year, depending on the SARS-CoV-2 impact on the population and varying difficulties implied in the norms that were adopted to embank the pandemic. Our objectives were to verify how the phenomenon of domestic violence has evolved and changed along 2020, and to clarify if these changes were correlated to the evolution of the pandemic.

**Methods:**

Though the analysis of the number of daily calls from women to the national anti-violence number and the parameters related to COVID-19 pandemic (daily cases, deaths, hospitalizations, and admissions in ICU), a positive correlation was found between daily deaths due to COVID-19 and the number of calls to the anti-violence number, while daily hospitalizations and admissions in ICU negatively correlated with calls of women reporting at the national anti-violence number.

**Results:**

The number of daily calls from women reporting at the national anti-violence number positively correlated with the number of quarantined people shifted of 30 days from the beginning of isolation at home, as well. We also analyzed temporal trends of daily calls from women to the national anti-violence number from 25th of February 2020 to 31st of December 2020.

**Conclusions:**

These findings demonstrate the importance of an active anti-violence telephone service and may help in developing a strategy to improve anti-violence facilities, especially during crises, such as specific sources of psychological support for women who have survived violence episodes.

## Introduction

The United Nations define violence against women as “any act of gender-based violence that results in, or is likely to result in, physical, sexual, or mental harm or suffering to women, including threats of such acts, coercion or arbitrary deprivation of liberty, whether occurring in public or in private [1]. Worldwide, nearly 30% of women have been subjected to physical and/or sexual violence by an intimate partner or non-partner sexual violence or both. In Europe, the prevalence estimates of lifetime intimate partner violence are around 22% [2]. Although being very difficult to identify environmental risk factors that may influence the prevalence of intimate partner violence, and since socio-demographic factors play an essential role in determining it, studies suggest that women are exposed to violence by their husbands no matter their educational level, nor their social status or economic conditions [3].

The literature has shown that in crisis situations (wars, natural disasters, or serious epidemics, regardless of the country concerned), intra-family violence increases [4, 5]. In the wake of Hurricane Katrina, which hit the USA in 2009, the prevalence of domestic violence had quadrupled; the physical violence suffered by women had almost doubled (4.2–8.3%) [6]. In addition to these findings, we can add that males tend to react to crisis through aggressiveness, with or without alcohol consumption [7].

Moving our focus to our still ongoing crisis, i.e., the COVID-19 pandemic, a meta-analysis has shown that noninfectious chronic disease patients, quarantined persons, and COVID-19 patients have a higher risk of depression and anxiety than other populations [8]. The spread of COVID-19 has caused a global economic crisis with inevitable repercussions on mental health, that need to be managed by both an increased government attention and an improvements of allocated resources [9]. Effects of the pandemic also concerned social phenomena such as family violence. A study conducted by Barchielli et al. [10] showed an increase in domestic violence during the lockdown period compared to the same period of 2019. The risk factors usually associated with family violence are exacerbated during epidemic periods (low income, fear of dying, social isolation, loss of reference points, loss of relatives, difficulties in accessing medical and social services, inability to run away, increased consumption of addictive substances, etc.) [10, 11]. Nevertheless, we should recall that partner violence can also happen in a familiar context, adding another burden to children witnessing aggressiveness episode, complicating their increasingly distressful world dictated by the COVID-19 pandemic [12, 13]. As a matter of fact, due to COVID-19 pandemic, calls to helplines have increased up to fivefold in some countries [14]. In Italy, a national anti-violence number (1522) was established in 2006 by the Ministry for Equal Opportunities, to counteract the increasing phenomenon of violence against women, in every form, both in families and in their extra-familiar life. Through victim support, the 1522 helpline gives women advice on how to report to national health services, in cooperation with law enforcement [15]. During 2019, the total number of daily calls to NAN was 8427, while it reached 15,128 during 2020, with a 79.5% increase. [16]

On these premises, our study has focused in analyzing the phenomenon of domestic violence reports from women along the 2020 pandemic in Italy. Our hypotheses are that the phenomenon of domestic violence during 2020 might have shown trend changes during 2020, and that possibly these changes could be correlated to different epidemiological aspects of the pandemic. Since joinpoint regression’s validity can be applied to trend analyses regarding psychological and pathological phenomena [17], we performed an analysis aimed at evaluating if during the 2020 pandemic, there has been a significant change in the number of daily anti-violence calls along months, depending on the varying social difficulties implied in the norms of social distancing that were imposed to embank the pandemic, and also on the personal life perspectives of people during a pandemic. Our objectives were to verify how the phenomenon of domestic violence has evolved and changed along 2020 and to clarify if these changes were correlated to specific factors linked to the evolution of the pandemic.

## Materials and methods

We collected data from the Italian Civil Protection Department of the Presidency of the Council of Ministers [18] regarding SARS-CoV-2 daily cases (DC), COVID-19 daily deaths (DD), daily hospitalizations (DH), daily intensive care unit (ICU) hospitalizations (D-ICU-H), daily number of people isolated at home (identifiable as “daily home quarantined,” DHQ), and daily dismissed patients (DDP), starting from 25th of February 2020 to 31st of December 2020, for a total of 310 days. The Italian National Institute of Statistics provided data of daily calls from women reporting at the National Anti-violence Number (NAN) during the year 2020 [16], which is the only official number for violence reports in Italy. Data were collected from each Italian Region and controlled by the Istituto Superiore di Sanità (ISS) and further verified by the Italian Ministry of Health and Civil Protection Department regarding quality of data, dataset elaboration and publication procedures on a daily basis [18].

We analyzed whether there could be a correlation between the COVID-related variables and the daily number of calls from women to Italian Anti-violence numbers.

### Statistical analysis

We used SPSS Statistics V25.0 software (IBM Corporation, Armonk, New York, 2016) for descriptive and inferential analysis, and Joinpoint Trend Analysis V4.9.0.0 Software [17] for trend analyses.

Pearson’s correlation test was conducted between the dimensional variables under study (i.e., SARS-CoV-2 DC, DD, DH, D-ICU-H, DHQ, and DDP, daily calls from women to NAN).

We analyzed temporal trends in the rate of violence reported calls using log-linear join-point segmented regression models, which identify points corresponding to statistically significant changes over time in the linear slope of the occurring trend [17, 19]. We used the daily rates of anti-violence calls as the dependent variable, assuming homoscedasticity and linearity, with log transformation for the assessment of significant changes in the trend based on the daily percent change (DPC). We applied an uncorrelated errors model. We set the minimum/maximum join-point number from 0 to 5 and used a permutation test with overall significance level set at *p* < 0.05.

## Results

The main aspects of the study variables, including daily calls to NAN and considered epidemiological variables, are resumed in the following Table 1, for each month of 2020 analyzed in the study.

**Table 1:** Descriptive statistics on daily calls to NAN and considered epidemiological variables

	Mean	Std. deviation	95% confidence interval for mean		Minimum	Maximum
			Lower bound	Upper bound		
Daily calls to the national anti-violence number						
February	18.0000	3.74166	12.0462	23.9538	13.00	22.00
March	27.3548	16.62638	21.2562	33.4534	6.00	68.00
April	70.9667	15.35889	65.2316	76.7018	34.00	94.00
May	62.8065	12.94455	58.0584	67.5545	36.00	89.00
June	44.3333	9.87275	40.6468	48.0199	22.00	68.00
July	54.7097	10.53627	50.8449	58.5744	39.00	85.00
August	42.4516	12.22794	37.9664	46.9369	18.00	67.00
September	32.2333	9.08080	28.8425	35.6242	18.00	52.00
October	30.9032	8.03474	27.9561	33.8504	12.00	50.00
November	49.5333	28.46502	38.9043	60.1623	18.00	147.00
December	41.0968	12.80457	36.4000	45.7935	20.00	72.00
Total	45.2355	19.82723	43.0197	47.4513	6.00	147.00
SARS-CoV-2 daily cases						
February	201.5000	82.50051	70.2233	332.7767	78.00	250.00
March	3376.2581	2050.62790	2624.0817	4128.4345	342.00	6557.00
April	3322.3667	934.78817	2973.3110	3671.4223	1739.00	4805.00
May	888.9032	435.58041	729.1310	1048.6754	300.00	1965.00
June	252.8000	87.18684	220.2439	285.3561	122.00	518.00
July	226.0000	62.56730	203.0501	248.9499	114.00	386.00
August	700.0645	415.25148	547.7490	852.3800	159.00	1462.00
September	1520.7667	249.67381	1427.5369	1613.9964	978.00	1912.00
October	11,761.5161	8967.40655	8472.2449	15,050.7874	2257.00	31,758.00
November	30,740.2000	6127.72091	28,452.0714	33,028.3286	16,377.00	40,902.00
December	16,416.5806	4153.82123	14,892.9467	17,940.2146	8585.00	24,099.00
Total	6807.5452	10,113.63892	5677.2833	7937.8070	78.00	40,902.00
SARS-CoV-2 daily deaths						
February	4.7500	2.50000	0.7719	8.7281	2.00	8.00
March	399.9677	315.13600	284.3749	515.5606	5.00	969.00
April	517.9667	132.82358	468.3695	567.5638	260.00	766.00
May	175.7419	91.02123	142.3551	209.1288	50.00	474.00
June	45.0667	27.14956	34.9289	55.2045	− 31.00	88.00
July	12.0645	6.89413	9.5357	14.5933	3.00	30.00
August	11.0323	27.44993	0.9635	21.1010	1.00	158.00
September	13.7000	5.18719	11.7631	15.6369	6.00	24.00
October	87.8710	75.38910	60.2180	115.5239	16.00	297.00
November	565.2667	173.54378	500.4644	630.0690	208.00	853.00
December	599.4516	167.32480	538.0764	660.8268	268.00	993.00
Total	239.1903	272.46231	208.7410	269.6397	− 31.00	993.00
Daily hospitalisations						
February	71.7500	46.72169	− 2.5946	146.0946	14.00	120.00
March	896.4839	532.25864	701.2498	1091.7179	103.00	2138.00
April	− 334.7667	415.43402	− 489.8923	− 179.6411	− 1107.00	269.00
May	− 379.4194	207.84542	− 455.6577	− 303.1810	− 802.00	− 79.00
June	− 176.5667	68.64084	− 202.1976	− 150.9358	− 299.00	− 30.00
July	− 12.0645	23.07226	− 20.5275	− 3.6015	− 65.00	21.00
August	18.4516	26.73928	8.6436	28.2597	− 22.00	83.00
September	58.6333	40.97980	43.3312	73.9354	− 15.00	131.00
October	481.2581	340.13595	356.4952	606.0209	45.00	1030.00
November	507.3667	534.94914	307.6134	707.1200	− 420.00	1331.00
December	− 323.7419	309.41428	− 437.2360	− 210.2479	− 1042.00	361.00
Total	74.3129	508.57939	17.4760	131.1498	− 1107.00	2138.00
Daily hospitalisations in ICU						
February	17.5000	17.52142	− 10.3805	45.3805	1.00	41.00
March	126.3871	58.99304	104.7483	148.0259	26.00	241.00
April	− 77.6333	40.27276	− 92.6714	− 62.5952	− 143.00	18.00
May	− 40.6129	35.02730	− 53.4610	− 27.7648	− 143.00	− 7.00
June	− 11.4000	11.48792	− 15.6897	− 7.1103	− 55.00	5.00
July	− 1.6774	3.45820	− 2.9459	− 0.4089	− 8.00	9.00
August	1.7097	3.13256	0.5606	2.8587	− 5.00	8.00
September	6.2000	4.42875	4.5463	7.8537	− 4.00	14.00
October	50.4194	37.11673	36.8048	64.0339	− 4.00	127.00
November	63.3667	57.27097	41.9813	84.7520	− 64.00	203.00
December	− 38.3548	27.20239	− 48.3328	− 28.3769	− 92.00	27.00
Total	8.1290	65.13190	0.8501	15.4079	− 143.00	241.00
Daily home quarantined						
February	95.2500	39.60114	32.2358	158.2642	59.00	131.00
March	1447.6452	1172.28502	1017.6475	1877.6428	− 337.00	4250.00
April	1209.6000	1083.44087	805.0365	1614.1635	− 1944.00	3050.00
May	− 1497.8387	1204.19318	− 1939.5403	− 1056.1371	− 6344.00	242.00
June	− 696.5000	455.28792	− 866.5073	− 526.4927	− 1690.00	− 38.00
July	− 87.5806	191.81949	− 157.9406	− 17.2207	− 607.00	230.00
August	420.3548	382.58320	280.0222	560.6875	− 19.00	1126.00
September	774.6667	227.62088	689.6716	859.6617	229.00	1082.00
October	9149.7097	7489.72644	6402.4558	11,896.9635	1097.00	25,470.00
November	13,998.7667	11,808.73084	9589.3141	18,408.2192	− 9525.00	32,195.00
December	− 6688.7097	6789.85952	− 9179.2503	− 4198.1691	− 26,557.00	5889.00
Total	1754.9290	7277.38797	941.6358	2568.2222	− 26,557.00	32,195.00
Daily home quarantined postponed by 30 days						
March	− 7714.1290	4300.80080	− 9291.6755	− 6136.5826	− 9999.00	255.00
April	1648.5333	1134.62978	1224.8556	2072.2111	− 337.00	4250.00
May	999.3548	1070.26824	606.7773	1391.9324	− 1944.00	3050.00
June	− 1595.5667	1149.83815	− 2024.9233	− 1166.2100	− 6344.00	242.00
July	− 636.1290	458.58302	− 804.3386	− 467.9194	− 1690.00	− 38.00
August	− 69.3548	190.99695	− 139.4131	0.7034	− 607.00	230.00
September	485.7333	377.04376	344.9429	626.5238	− 19.00	1126.00
October	832.0323	283.76381	727.9469	936.1177	229.00	1503.00
November	10,752.8667	7804.01895	7838.7981	13,666.9352	1097.00	25,711.00
December	11,289.1290	13,429.23466	6363.2460	16,215.0121	− 18,311.00	32,195.00
Total	1433.8032	7441.76141	602.1403	2265.4662	− 18,311.00	32,195.00

The Pearson correlation coefficient showed that COVID-19 DD were positively correlated with the number of daily calls to NAN (*r* = 0.285; *p* < 0.001), but DC of SARS-CoV-2 were not correlated with the number of daily calls to NAN (*r* = − 0.063; *p* = 0.271). Furthermore, a negative correlation was found between DH (*r* = − 0.450; *p* < 0.001), D-ICU-H (*r* = − 0.524; *p* < 0.001), and DHQ (*r* = − 0.231; *p* < 0.001) and the number of daily calls from women reporting at NAN. Nevertheless, the number of quarantined people who had spent 30 days isolated at home (evicted from DHQ postponed by 30 days) positively correlated with the number of daily calls from women reporting at NAN (*r* = 0.339; *p* < 0.001) (Table 2)*.*

**Table 2:** Correlation table between SARS-CoV-2 DC, COVID-19 DD, DH, D-ICU-H, DHQ and DHQ postponed by 30 days, and the number of daily calls to NAN

	Number of daily calls to national anti-violence number
SARS-CoV-2 daily cases	
Pearson correlation	− 0.063
Sig. (2-tailed)	0.271
SARS-CoV-2 daily deaths	
Pearson correlation	0.285**
Sig. (2-tailed)	**< 0.001**
Daily hospitalisations	
Pearson correlation	− 0.450**
Sig. (2-tailed)	**< 0.001**
Daily hospitalisations in ICU	
Pearson correlation	− 0.524**
Sig. (2-tailed)	**< 0.001**
Daily home quarantined	
Pearson correlation	− 0.231**
Sig. (2-tailed)	**< 0.001**
Daily home quarantined postponed by 30 days	
Pearson correlation	0.339**
Sig. (2-tailed)	**< 0.001**

Bold characters are meant to underline significance of* p* < 0.05

**Fig. 1:**
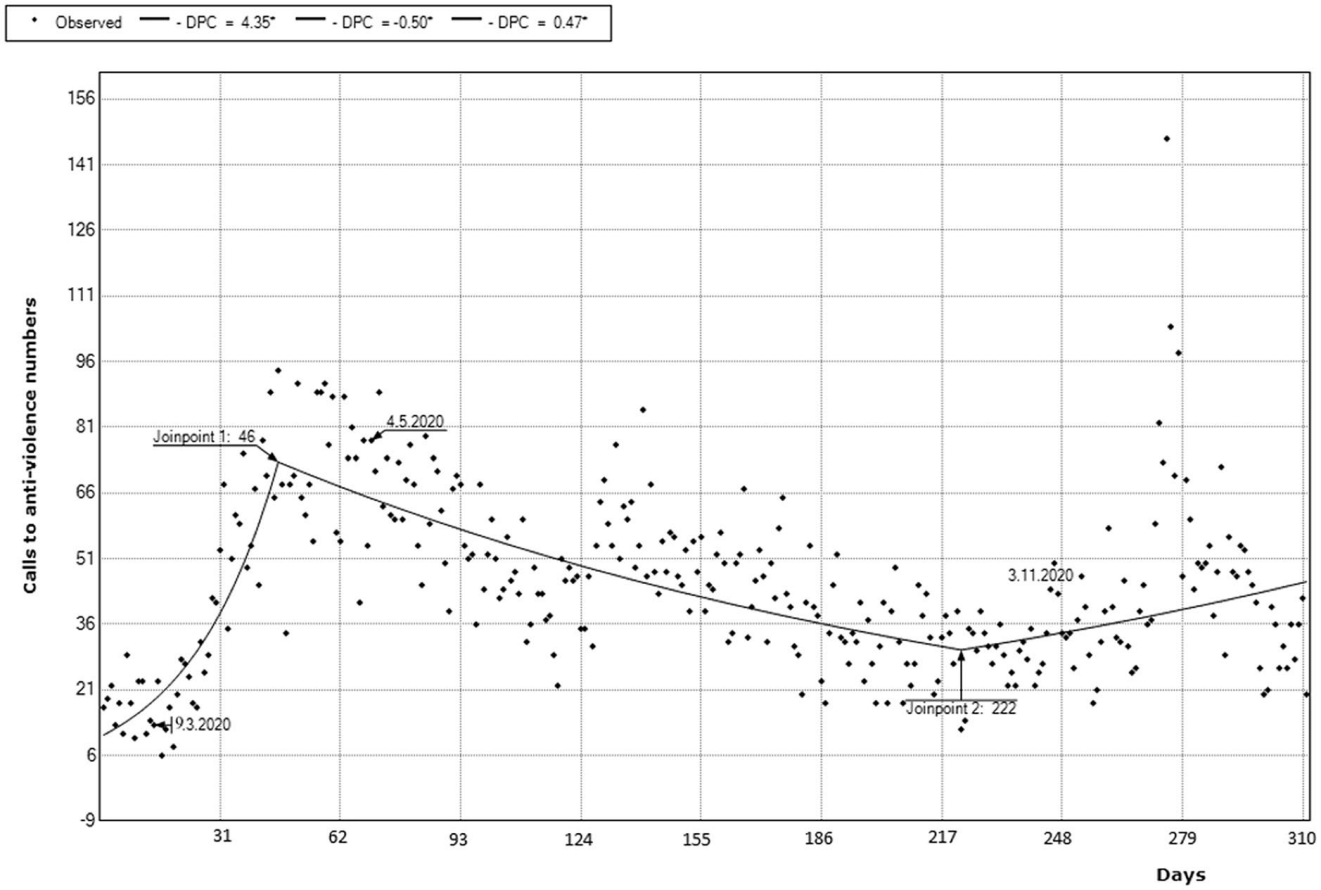
Daily number of calls to the national anti-violence number (NAN) from 25th of Feb. 2020 to 31st of Dec. 2020. The three dates highlighted correspond to 9th of March 2020, the beginning of the first lockdown; 4th of May 2020, the ending of the 1st lockdown; 3rd of November 2020, the introduction of night curfew. *Indicates that the Daily Percent Change (DPC) is significantly different from zero at the alpha = 0.05 level. Final selected model: 3 Joinpoints

The join-point regression of the daily calls to the anti-violence number showed a significant 2-joinpoint model with three significant segments, the first between days 1 (25 February) and 46 (10 April) (DPC = 4.4%; *t* = 11.2; *p* < 0.001), the second between 46 and 222 (DPC = − 0.5%; *t* = − 10.2; *p* < 0.001), the third between 222 (3 October) and 311 (31 December) (DPC = 0.5%; *t* = 3.4; *p* = 0.001) as shown in Table 3 and Fig. 1.

**Correlation is significant at the 0.01 level (2-tailed).

**Table 3:** Trends of 2020 daily calls to the Italian anti-violence number, from 25th February 2020 to 31st December 2020

Segment	Lower endpoint	Upper endpoint	DPC	Lower CI	Upper CI	Test statistic (*t*)	Prob > |*t*|
1	1 (25th of February 2020)	46 (10th of April 2020)	4.4*	3.6	5.1	11.2	< 0.001
2	46 (10th of April 2020)	222 (3rd of October 2020)	− 0.5*	− 0.6	− 0.4	− 10.2	< 0.001
3	222 (3rd of October 2020)	310 (31st of December 2020)	0.5*	0.2	0.7	3.4	0.001

## Discussion

This is the first descriptive study focused on the trend of anti-violence calls for help to NAN in Italy during the SARS-CoV-2 pandemic in 2020.

An initial intuitive expectation would have been to find a positive correlation between an essential parameter such as SARS-CoV-2 DC and the daily number of calls to NAN. Nonetheless, we have found that this correlation existed with DD. We can speculate that one of the main psychological burdens of COVID-19 in Italy has been the coping with the high number of DD. A recent study’s participants [20] described feeling unsupported when confiding in others about their experiences of loss and grief during COVID-19. This latter, while experiencing these feelings, may have led to discussions which may have precipitated in aggressive behaviors.

A peculiar result of our study is the negative correlation between daily number of calls to NAN and DH and D-ICU-H. A possible explanation could be that when a whole family is isolated and sick at home, higher interpersonal distress may lead to difficulties in peacefully relate to one another. The hospitalization of a family member might indirectly resolve a stressful situation, leading one individual far from both their family and also from any potential relational issue.

The most striking finding of our study is the correlation between daily number of calls to NAN and the number of quarantined people shifted at 30 days at home. It can be supposed that a prolonged quarantine, leading to prolonged contact with one another, may act as a risk factor for developing family conflicts, as one needs not only to cope with their own problems, but also with the others’. The literature has shown that quarantine has been strongly associated with anxiety [21]. In other words, we can assume that those who were forced to spend a long time isolated at home had to cope not only with their own increasing anxiety toward what future was holding, but also with their relatives’/cohabitants, leading to a tense home environment. In a recent study, anxiety was found to be the second risk factor for the perpetration of intimate partner violence since the onset of COVID-19, while feelings of loneliness were the first [22].

Regarding Fig. 1, both joinpoints seem to be related to key moments of the pandemic, during which we can hypothesize that the general population was creating expectations for their future. Joinpoint 1 corresponds to the 34th day after the beginning of the first lockdown, the 10th of April 2020. During this day, the Italian PCM allowed for a partial re-opening of some commercial activities, and it indicated that the 3^rd^ of May would have been the end of the national lockdown. We can hypothesize that this helped people find some hope in the future, reducing general frustration. On the other hand, since at that point of the pandemic people had been isolated for a maximum of 34 days, we can also confirm the speculation that social isolation (and worse, quarantine) led to a home environment which predisposed to aggressive behavior as a mechanism to cope with the difficulties of that time, as a peak of calls was reached anyway on that joinpoint.

Regarding joinpoint 2, corresponding to the 222nd day analyzed, and to the 3rd of October 2020, a pandemic period in which a progressive worsening of the scenario was happening. The perspective of a curfew was becoming clearer and had already been foreseen by the PCM, leading again to the loss of safety and hope that people had partially found again during the previous months. For these reasons, we can speculate that the increase (or decrease) in calls to the national anti-violence number depended not only on the official dates of PCM measures to contain the pandemic, but also on the expectations that the population had regarding future.

Two remarks worth of mentioning regard the psychological status of women who were victims of violence during COVID-19, i.e., one of the main concerns that led to the development of this study. The first remark points out the need of a strong psychological support for those who reported violence: a study conducted in Spain among professionals delivering this type of support reported indeed that during the confinement, there was an increased demands for psychological support, primarily related to the need to speak and be listened, but also to the need for strategies for cohabitating with the aggressors [23].

The second issue that needs to be underlined is what Burke et al. [24] have described as a key behavioral change triggered by the pandemic, through the creation of a context in which women feel the need for ending a violent relationship, including preparing for leaving and the use of safety strategies against perpetration of violence episodes. This can be explained also on the light that women who report violence are not usually at their first victim episode, meaning that they probably have suffered violence before [25].

Taking into consideration these thoughts on the light of our findings, the need of an improvement of both psychological and social support for women victims of violence becomes urgent and mandatory.

### Limitations

Our study did not take into consideration some aspects of human differences of women referring to NAN, such as sociodemographic variables and type of violence reported. However, the main objective of the study was to provide a description of the general trend of the phenomenon during the year 2020, and ISTAT [16] and PCMDPO [15] showed the main aspects of human differences.

## Conclusions

First, our study has demonstrated the importance of a national anti-violence number as a central service of listening for women dealing with this social and health problem. Moreover, a correlation between the burden of deaths of COVID-19 in Italy and the increase in women reports to the anti-violence number was showed. Nonetheless, hospitalizations due to COVID-19 were inversely correlated with violence episode reports.

Prolonged quarantine measures were also positively correlated with the number of calls from women reporting to the national anti-violence number. Taken together, these findings highlight the need to establish more detailed programs for the prevention of violence against women, and, more specifically, for the psychological support of women who have survived violence episodes.
